# Plasma Exchange for Hantavirus Pulmonary Syndrome: A Mechanistic Case for an Untested Intervention

**DOI:** 10.1002/jca.70187

**Published:** 2026-09-28

**Authors:** Menatalla Nadim, Yasamin Mirzabeigi, Yamac Akgun

**Affiliations:** ^1^ Department of Pathology and Laboratory Medicine University of Miami Miller School of Medicine Miami Florida USA

**Keywords:** cytokine storm, endothelial injury, hantavirus pulmonary syndrome, plasma, therapeutic plasma exchange

## Abstract

Hantavirus pulmonary syndrome (HPS) carries a case fatality rate of 35%–40% and has no approved pharmacologic treatment. Its pathophysiology is rooted in the plasma compartment: cytokines, viral antigens, immune complexes, and platelet‐binding glycoproteins circulate as drivers of immunopathologic endothelial injury, noncardiogenic pulmonary edema, and cardiogenic depression. Therapeutic plasma exchange (TPE) achieves bulk clearance of plasma‐borne mediators and is established by the American Society for Apheresis (ASFA) for numerous indications that share an analogous mechanistic rationale. This commentary argues that the immunopathology of HPS maps onto the therapeutic targets of TPE, that any intervention would need to be timed to the early cardiopulmonary phase, and that the coagulopathic dimension of the disease provides a mechanistic argument for incorporating plasma into the replacement strategy. We deliberately situate this rationale against the uneven record of TPE in diseases of comparable pathophysiology, in which the intervention has been effective in some settings and without demonstrable benefit in others. The recent Andes virus outbreak aboard the cruise ship MV Hondius, which produced a multinational cluster of severe cardiopulmonary cases, prompted us, as transfusion medicine and apheresis physicians, to articulate this hypothesis. We emphasize that these arguments are mechanistic and hypothesis‐generating rather than evidence‐based: no clinical data yet support TPE in HPS, and systematic outcome reporting, ideally through a multicenter case series, would be required before any conclusion about efficacy could be drawn.

## 1 Introduction

Hantavirus pulmonary syndrome (HPS), caused primarily by Sin Nombre virus in North America and Andes virus in South America, remains one of the most lethal respiratory infections encountered in the Western Hemisphere. Since its identification following the 1993 Four Corners outbreak, HPS has been characterized by a case fatality rate approaching 35%–40%, a figure that has not materially improved in three decades despite advances in critical care [1, 2]. No antiviral agent has demonstrated efficacy in controlled trials. Ribavirin, the most extensively studied candidate, failed to reduce mortality in a prospective randomized trial and is not recommended for treatment of established HPS [3]. Management remains entirely supportive: mechanical ventilation for respiratory failure, vasopressors for cardiogenic and distributive shock, and extracorporeal membrane oxygenation (ECMO) as a rescue strategy in refractory cases [4, 5].

The persistent gap between the severity of this disease and the poverty of its treatment options invites reexamination of established extracorporeal therapies. Therapeutic plasma exchange (TPE), a procedure central to transfusion medicine practice and codified in the American Society for Apheresis (ASFA) guidelines for more than 40 indications, has never been formally evaluated or published as a treatment for HPS [6]. This commentary argues that the immunopathology of HPS maps onto the mechanistic rationale for TPE, that the plasma compartment represents a plausible site of actionable pathology, and that the replacement fluid strategy in this context deserves specific attention from a transfusion medicine perspective. We advance these points as a hypothesis to be tested rather than as a recommendation for immediate practice.

Two considerations prompted this commentary. The first is mechanistic and is developed below. The second is circumstantial. The 2026 outbreak of Andes virus infection aboard the cruise ship MV Hondius, which generated a multinational cluster of hantavirus cardiopulmonary cases with a case fatality ratio within the range historically reported for the disease, returned clinical and public health attention to a syndrome whose therapeutic options have remained essentially static for three decades [7]. We write as transfusion medicine and apheresis physicians rather than as clinicians with first‐hand experience managing HPS, and we are deliberately explicit about that vantage point. Our aim is to frame a mechanistically coherent hypothesis from the perspective of the extracorporeal therapies we practice, so that centers that do encounter these patients, whether in sporadic cases or in the next cluster, are positioned to evaluate and report outcomes systematically.

## 2 Pathophysiology: The Plasma Compartment as Battlefield

Understanding why TPE is mechanistically rational for HPS requires understanding why HPS kills (Figure 1). Hantaviruses infect pulmonary microvascular endothelial cells without inducing direct cytopathic effect [8]. Infected endothelial cells remain architecturally intact; it is the immune response mounted against viral antigens displayed on their surface that destroys the vascular barrier. Cytotoxic CD8+ T lymphocytes, recruited in disproportionate numbers, drive immunopathologic endothelial injury through perforin and granzyme‐mediated mechanisms [9]. The result is not pneumonia in the conventional sense but a diffuse increase in pulmonary vascular permeability, causing protein‐rich fluid to flood the alveolar compartment. The clinical syndrome that follows, noncardiogenic pulmonary edema progressing to refractory hypoxemia within 24–48 h of the cardiopulmonary phase onset, reflects this mechanism precisely [2].

**FIGURE 1:**
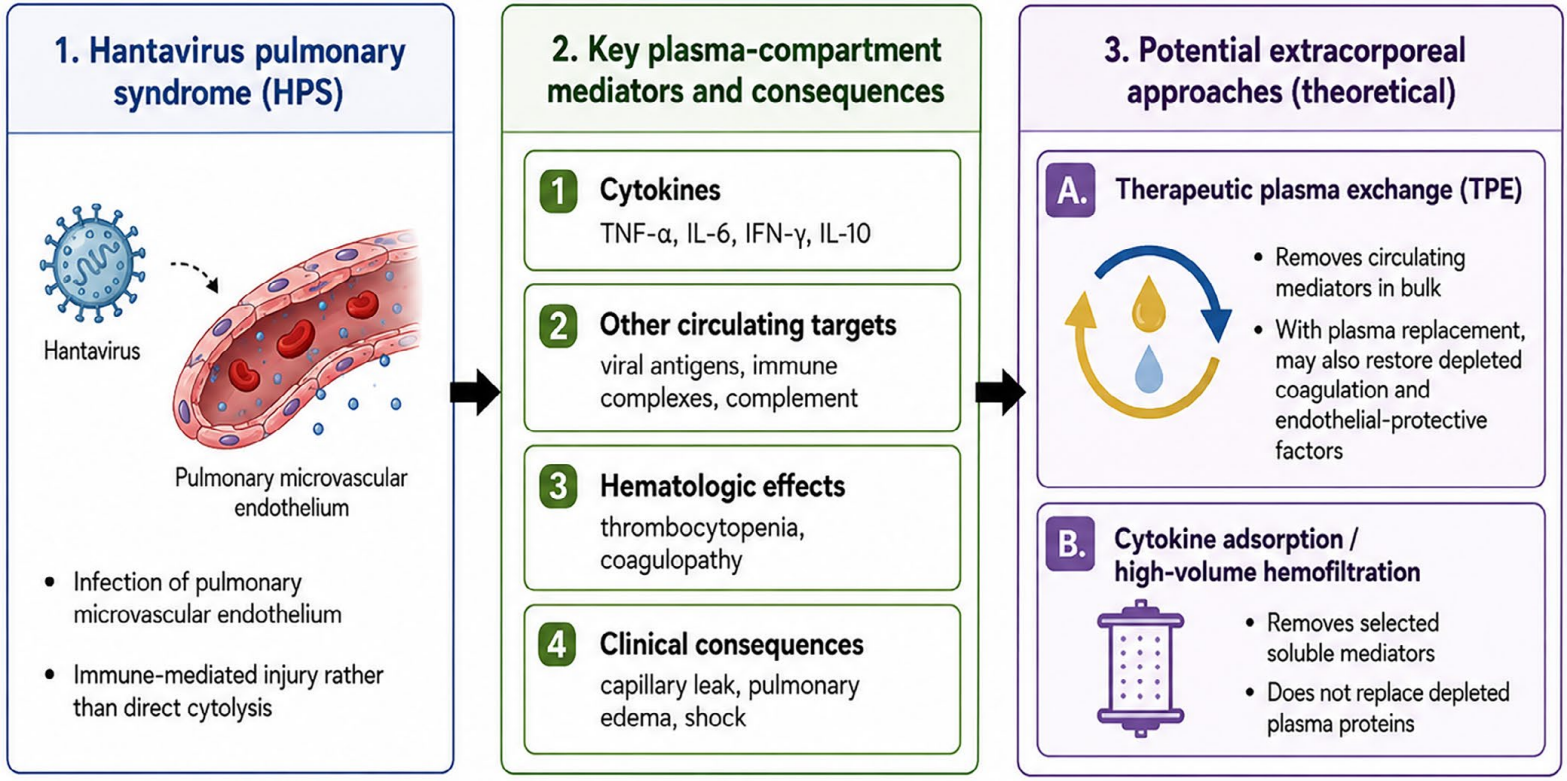
Conceptual rationale for plasma‐directed extracorporeal therapy in hantavirus pulmonary syndrome. TPE and cytokine‐directed extracorporeal therapies are presented as theoretical, hypothesis‐generating approaches; clinical efficacy remains unproven.

The cytokine milieu in HPS is the proximate driver of this process. Circulating levels of tumor necrosis factor‐alpha (TNF‐alpha), interleukin‐6 (IL‐6), interleukin‐10 (IL‐10), and interferon‐gamma are markedly elevated in patients with severe disease, correlating with clinical deterioration [10, 11]. These mediators perpetuate endothelial activation, upregulate adhesion molecule expression, and amplify capillary leak beyond the initial viral trigger. Simultaneously, thrombocytopenia, universally observed in HPS, reflects platelet consumption, impaired thrombopoiesis, and direct binding of hantavirus glycoproteins to platelet integrin alpha‐IIb‐beta‐3 [12]. Viral antigens and immune complexes circulate during the acute phase, potentially sustaining toll‐like receptor and innate immune activation even as viremia begins to clear.

The myocardial depression characteristic of HPS adds a cardiogenic component to what is otherwise a distributive and permeability‐driven shock syndrome. Depressed left ventricular ejection fraction in the acute phase is well described and reflects the effects of circulating mediators on myocardial contractility rather than ischemia or direct viral invasion [5, 13]. This low cardiac output state, superimposed on alveolar flooding from capillary leak, produces the particularly lethal hemodynamic phenotype that distinguishes HPS from other viral pneumonias and has prompted the preferred designation hantavirus cardiopulmonary syndrome (HCPS).

The central observation for the transfusion medicine audience is this: the pathogenic burden in HPS is not primarily cellular or structural. It is humoral. Cytokines, viral antigens, immune complexes, activated complement fragments, and dysfunctional platelet‐binding viral glycoproteins all circulate in plasma. The plasma compartment is both the propagation medium and the therapeutic target.

## 3 Mechanistic Rationale for Therapeutic Plasma Exchange

TPE removes one to one and a half plasma volumes per session, clearing approximately 63%–78% of any given plasma constituent in a single procedure [14]. In diseases characterized by pathogenic circulating mediators, this degree of bulk clearance exceeds what pharmacologic suppression or renal replacement therapy can achieve on a comparable timescale. The ASFA guidelines assign Category I or II designations to disorders spanning autoimmune, hematologic, and neurologic disease on exactly this basis [6]. Applied to HPS, TPE would be expected to achieve several mechanistic objectives simultaneously.

Cytokine clearance is the most immediate rationale. TNF‐alpha, IL‐6, and interferon‐gamma are predominantly plasma‐borne mediators in the molecular weight range of 17 to 26 kDa, well within the clearance profile of centrifugal plasma separation. A single 1.0 plasma volume exchange reduces circulating cytokine levels by approximately 50 to 65% before endogenous resynthesis begins [15]. In a disease where the severity and duration of the cytokine storm directly predict mortality, even a transient reduction in circulating mediator burden could plausibly interrupt the immunopathologic cascade before irreversible lung injury is fully established. This therapeutic window, corresponding roughly to Days 4–8 of illness when the cardiopulmonary phase begins, is when any intervention would need to be timed.

Viral antigen and immune complex removal represents a distinct and complementary rationale. Hantavirus nucleocapsid antigen and glycoprotein fragments circulate during the acute viremic phase, potentially sustaining innate immune activation through pattern recognition receptor engagement. Immune complexes formed between viral antigens and early antibody responses can deposit in vascular beds and amplify complement‐mediated endothelial injury. TPE removes both the antigen burden and the complexes formed against it, reducing the substrate for ongoing complement activation.

Restoration of ADAMTS13 and endothelial‐protective proteins is a mechanism that has received insufficient attention in the emerging infection literature but is relevant here. Severe inflammatory states consume ADAMTS13, the von Willebrand factor‐cleaving protease whose deficiency underlies thrombotic microangiopathy. Whether HPS is accompanied by clinically significant ADAMTS13 depletion has not been formally studied, but the consumptive coagulopathy and platelet dysfunction observed in the disease create a phenotype that could benefit from restoration of this and other regulatory plasma proteins [16].

## 4 Replacement Fluid Strategy: A Transfusion Medicine Perspective

The choice of replacement fluid in TPE for HPS deserves specific consideration and distinguishes this hypothetical indication from the majority of ASFA‐guided diseases in which albumin alone suffices. In most established indications, albumin replacement is preferred because it avoids the infectious and allergic risks associated with plasma products and adequately restores oncotic pressure. In HPS, however, the coagulopathic dimension of the disease argues for a different calculus.

Patients in the cardiopulmonary phase of HPS frequently exhibit prolonged prothrombin times, elevated fibrin degradation products, and reduced fibrinogen, reflecting a consumptive coagulopathic process superimposed on severe thrombocytopenia [2, 16]. In this setting, albumin‐only replacement across serial exchanges would be expected to accelerate depletion of residual coagulation factors by dilution, potentially worsening an already compromised hemostatic capacity. This concern provides a mechanistic argument for using plasma for all or a substantial portion of the exchange volume in HPS. We stress, however, that this remains a mechanistic argument and not an evidence‐based one: albumin replacement has not been shown to be ineffective or harmful in HPS, no comparative data exist, and the choice of replacement fluid in any individual patient must weigh the theoretical hemostatic benefit of plasma against its infectious, allergic, and TRALI‐associated risks.

Beyond coagulation factor repletion, plasma provides alpha‐2‐macroglobulin (a broad‐spectrum protease inhibitor and cytokine carrier), protein C and antithrombin (anti‐inflammatory and anticoagulant mediators consumed during critical illness), and endothelial‐protective proteins that are stripped away by the inflammatory milieu. This argument parallels the rationale for plasma replacement in TPE for thrombotic microangiopathy, where ADAMTS13 repletion is the explicit therapeutic goal [6]. In HPS, the goal would be broader, but the logic is analogous: replace what the disease process has consumed while simultaneously removing what it has generated in excess. The strength of this analogy, however, should not be overstated in the absence of any HPS‐specific data.

Practical considerations regarding ABO compatibility and the risk of transfusion‐related acute lung injury (TRALI) in a patient with already‐compromised pulmonary reserve require careful attention and close coordination between the apheresis service and the intensive care unit. Because the residual risk of transfusion‐transmitted viral infection from modern plasma products is already very low, the incremental safety benefit of pathogen‐reduced plasma in this context is modest; where such products are available, however, they may offer an additional margin of safety that is reasonable to prefer given the setting of active viral infection [17]. The volume dynamics of a 1.0–1.5 plasma volume exchange in a hemodynamically unstable patient with diminished cardiac reserve mandate continuous hemodynamic monitoring throughout the procedure and may require vasopressor support during the exchange itself.

## 5 Alternative Extracorporeal Strategies: Cytokine Adsorption and Hemofiltration

TPE is not the only extracorporeal option capable of lowering the circulating mediator burden in HPS, and any argument for it must account for the alternatives. Selective cytokine adsorption columns, such as those incorporating porous polymer beads, and high‐volume hemofiltration both reduce plasma cytokine concentrations and have been described in cytokine‐driven critical illness, including a reported case of hantavirus cardiopulmonary syndrome treated with high‐volume hemofiltration [18, 19]. These modalities carry real advantages in the coagulopathic HPS patient: they do not require large volumes of plasma, they avoid the transfusion exposure and TRALI risk inherent to plasma replacement, and adsorption can often be integrated into an existing continuous renal replacement circuit. Their principal limitation relative to TPE is that they are subtractive only. Adsorption and hemofiltration remove cytokines within a defined size range but do not efficiently clear larger immune complexes and do not replete the regulatory and hemostatic plasma proteins that the disease consumes. TPE is distinctive precisely because it couples broad removal with simultaneous restoration of plasma composition, the feature most relevant to the consumptive coagulopathy of HPS. We do not regard these approaches as mutually exclusive; in a given patient, adsorption, hemofiltration, and TPE could prove complementary, and the relative merits of each remain entirely untested in this disease.

## 6 Precedent and Positioning

TPE has not been used for HPS in any published report, but extracorporeal cytokine removal has. High‐volume continuous hemofiltration (HVHF) was applied to a patient with HCPS and refractory cardiogenic shock in a 2016 Chilean case report, with hemodynamic improvement within hours of initiation [18]. The authors attributed the benefit specifically to cytokine clearance across the hemofilter membrane. If cytokine removal via CRRT can influence the HPS shock state, the case for TPE, which achieves cytokine clearance with greater efficiency per unit time and simultaneously restores plasma protein composition, is at least worth examining. A single case report, however, cannot establish efficacy for either modality.

Convalescent plasma has also been examined in the HPS context, with a small interventional study demonstrating a borderline‐significant reduction in case fatality rate when administered early in the viremic phase [20]. Convalescent plasma and TPE are mechanistically distinct: the former provides neutralizing antibodies against viral antigens, the latter removes pathogenic circulating mediators. They could in principle be complementary in a sequential protocol, with TPE addressing the immunopathologic phase and convalescent plasma targeting viremia in the days preceding it. The analogy to antibody replacement following TPE in myasthenia gravis is imperfect but instructive.

More broadly, TPE has been applied to cytokine‐storm syndromes of diverse etiology including hemophagocytic lymphohistiocytosis, macrophage activation syndrome, and severe COVID‐19, where the pathophysiology shares important features with HPS: immunopathologic endothelial injury, consumptive coagulopathy, and cytokine‐driven organ failure [21, 22]. The mechanistic parallels support extrapolation of the TPE rationale to HPS, while acknowledging that the viral trigger and the specific immune effector populations differ.

This mechanistic optimism must be tempered by the uneven record of TPE across diseases whose pathophysiology superficially resembles that of HPS, because plausibility has repeatedly failed to predict benefit. On the favorable side, TPE is unambiguously life‐saving in thrombotic thrombocytopenic purpura, and it has produced apparent benefit in selected reports of hemophagocytic lymphohistiocytosis, macrophage activation syndrome, and catastrophic antiphospholipid syndrome, all disorders defined by circulating mediators and endothelial injury [21]. On the unfavorable side, sepsis and septic shock, the archetypal cytokine‐storm and endothelial‐injury syndrome, illustrate the opposite lesson. Despite a mechanistic rationale essentially identical to the one advanced here, and despite a small randomized trial suggesting a mortality reduction [15], the broader evidence for TPE in adult sepsis remains insufficient to establish benefit, and the intervention is not part of standard management [23]. Controlled evaluation of TPE in severe COVID‐19, another cytokine‐driven endothelial disease, similarly failed to demonstrate a consistent mortality benefit despite encouraging observational signals, and extracorporeal interventions in acute respiratory distress syndrome of non‐hantaviral origin have not yielded reproducible benefit. The recurring lesson is that a compelling mechanistic overlap does not guarantee clinical efficacy. We therefore present the HPS rationale explicitly as a hypothesis to be tested, not as an extrapolation to be assumed.

The ASFA guidelines currently list no hantavirus indication, reflecting the absence of published clinical data rather than a mechanistic objection [6]. It is important to be realistic about what would be required to change this. ASFA categories are not assigned on a linear scale in which any positive report advances an indication; the assigned category and grade of recommendation reflect the quality and consistency of the underlying evidence base. A single case, or even one or two favorable case reports, would be very unlikely to establish a new fact sheet, particularly given the strong bias toward publication of positive outcomes discussed below. A more realistic path would be systematic, prospectively structured reporting from a multicenter case series that captures cytokine profiling and coagulation parameters before and after exchange, together with adverse events and the outcomes of patients who did not respond. Whether such data would ultimately meet the threshold for formal ASFA consideration is a determination for the ASFA writing committee and the editorial board, not one that the present authors can assert.

## 7 Limitations

Several limitations bound this commentary. First and most fundamental, it is entirely hypothetical. No published experience with TPE in HPS exists, and the authors have no first‐hand experience treating this disease. Every argument advanced here is mechanistic and must be treated as hypothesis‐generating rather than as guidance for practice. Second, the absence of published TPE experience in HPS cannot be assumed to mean the intervention has never been attempted. Unsuccessful or negative attempts in individual patients are disproportionately likely to go unreported, since studies with positive clinical results are published substantially more often than those with negative results [24]. The apparent silence of the literature may therefore reflect publication bias as much as a true absence of experience, and it would be a mistake to read the lack of negative reports as reassurance. Third, the mechanistic parallels we draw to other cytokine‐driven diseases cut both ways, as the mixed record reviewed above makes clear, and the specific viral trigger and immune effector populations of HPS differ from those of the comparator conditions. Fourth, the replacement fluid argument rests on the coagulopathic phenotype of HPS and on analogy to other indications; it has not been tested, and albumin has not been shown to be inferior. We present these arguments in full awareness of these constraints, and precisely because of them we frame the appropriate next step as careful observation and systematic reporting rather than routine application.

## 8 Conclusion

HPS remains a disease without effective treatment, and its pathophysiology is rooted in the plasma compartment, the therapeutic target of TPE. On mechanistic grounds, the case for evaluating TPE in HPS is at least as strong as it has been for several indications that were studied before robust evidence existed, and the replacement fluid strategy can in principle be tailored to the coagulopathic and endothelial‐depleting dimensions of the disease. At the same time, the mixed results of TPE in mechanistically similar conditions, the real possibility of unpublished negative experience, and the complete absence of clinical data in HPS itself all counsel caution.

What is missing is not a plausible rationale. What is missing is evidence. Apheresis physicians, transfusion medicine specialists, and intensivists who encounter HPS in patients failing conventional support might reasonably consider TPE as a mechanistically rational rescue option, but only with explicit acknowledgment of its unproven status, careful attention to procedural risk in hemodynamically fragile patients, and a commitment to reporting outcomes systematically, including the outcomes of patients who do not respond. The time to design that evaluation is between outbreaks, not in the middle of one.

## Funding

The authors have nothing to report.

## Conflicts of Interest

The authors declare no conflicts of interest.

## Data Availability

Data sharing not applicable to this article as no datasets were generated or analyzed during the current study.
